# Multidimensional Prosodic and Semantic Coherence Modeling for Mandarin Mild Cognitive Impairment Detection

**DOI:** 10.3390/bioengineering13070748

**Published:** 2026-06-26

**Authors:** Rongyu Li, Meihong Wu

**Affiliations:** 1 School of Informatics, Xiamen University, 422 Siming South Road, Xiamen 361005, China; rongyul@andrew.cmu.edu; 2 Hearing and Speech Laboratory, Xiamen University, 422 Siming South Road, Xiamen 361005, China

**Keywords:** mild cognitive impairment (MCI), multimodal fusion, deep learning, speech analysis, prosodic features

## Abstract

Early detection of Alzheimer’s disease (AD) and mild cognitive impairment (MCI) remains critically important, yet conventional neuroimaging and biomarker-based approaches are costly, invasive, and poorly scalable for population screening. Speech offers a non-invasive, cost-effective alternative cognitive biomarker, but existing systems rarely integrate its multiple linguistic dimensions. We present Multi-Spec MCI-Net, a multimodal framework for HC/MCI classification that jointly models three complementary speech representations: token-level semantics via dVAE and BERT operating on Mel spectrograms; temporal prosodic dynamics via a 1D-CNN with attention; and discourse-level semantic coherence via a graph convolutional network. A gated fusion mechanism adaptively weights these modalities, yielding clinically interpretable predictions tailored to individual phenotypic profiles. Evaluated on the Chinese NCMMSC2021_AD challenge dataset and the DementiaBank Mandarin subset, the model achieves 89.29% accuracy and 0.9584 ROC AUC on NCMMSC2021_AD, with 92.31% MCI recall—critical for minimizing false negatives in screening contexts. Evaluation on the combined NCMMSC2021_AD and DementiaBank Mandarin dataset attains 77.46% accuracy and 0.8280 AUC, demonstrating robustness across spontaneous dialog and picture description tasks. Ablation studies confirm that multimodal fusion outperforms the semantic-only baseline by 5.16 percentage points, with each branch contributing non-redundant diagnostic information. These results establish an effective, interpretable approach for scalable, speech-based early MCI screening.

## 1. Introduction

Alzheimer’s disease (AD) is the most prevalent neurodegenerative disorder globally, accounting for the majority of dementia cases [1,2,3,4]. Mild cognitive impairment (MCI) is widely recognized as a transitional stage between normal aging and AD, with a substantial proportion of MCI patients progressing to AD over time [5,6,7,8]. Given that early intervention yields superior clinical outcomes compared to late-stage treatment, reliable early screening of MCI is of paramount clinical importance.

At present, common diagnostic approaches include neuropsychological tests, neuroimaging, and biomarker analysis. Neuropsychological tools such as the MMSE and MoCA are widely used, but they are strongly influenced by education level and can be subjective in early-stage screening [9,10]. Neuroimaging methods such as MRI and PET are more accurate, yet they are expensive and require specialized equipment and personnel [11,12]. Biomarker analysis, including CSF markers such as Aβ42 and p-Tau and blood markers such as plasma p-Tau217, is promising but may be invasive, costly, or not yet widely available [13,14,15]. Overall, these methods are difficult to apply in large-scale early screening.

In contrast, speech-based detection offers significant advantages as a non-invasive and cost-effective alternative, with considerable clinical appeal owing to the fact that cognitive decline frequently leaves quantifiable traces across prosodic, semantic, and syntactic domains [16,17,18,19,20,21,22,23]. For example, patients may produce more and longer pauses, slower speech, and reduced fundamental frequency (F0) variability at the prosodic level [20,22]; at the semantic level, they may show weaker lexical diversity, less coherent discourse, and frequent topic shifts [18,19]; and at the syntactic level, their utterances may become simpler or more fragmented [21,23]. Collectively, these alterations indicate that natural speech encodes robust, informative biomarkers for detecting cognitive decline.

Recent studies have shown that speech-based methods can detect cognitive impairment with promising performance [24,25,26,27,28,29,30]. However, several limitations remain. Many multimodal systems still rely on simple feature fusion and do not fully model the complementarity between modalities. Semantic modeling is often limited to lexical or syntactic statistics, while semantic coherence is not sufficiently explored. Prosodic modeling is also commonly reduced to summary statistics, which may miss subtle temporal dynamics. Furthermore, while many deep learning models achieve good accuracy, they often operate as “black boxes” with limited interpretability.

Crucially, beyond these engineering challenges, the field is pervasively constrained by a “single-language bias” and a lack of cross-lingual validation. The vast majority of state-of-the-art models heavily rely on monolingual datasets—predominantly English corpora such as DementiaBank Pitt and the ADReSS challenge datasets. This data bias implicitly forces existing architectures to overfit the underlying acoustic patterns and syntactic topologies of stress-timed languages. However, different language systems exhibit fundamental typological differences in cognitive neurolinguistic mapping. For instance, Mandarin Chinese, as a typical tone language, relies on the micro-dynamic variations of fundamental frequency not only for paralinguistic affect and prosody but also as a direct phonemic determinant of core lexical semantics. Consequently, speech biomarkers excavated in a monolingual English context cannot be smoothly transferred to other language families. This lack of systematic cross-lingual validation masks the vulnerability of deep neural networks to heterogeneous languages and remains a core barrier to deploying speech-based diagnostic tools in diverse global clinical settings. Therefore, constructing a comprehensive multimodal baseline framework tailored to the unique articulatory and cognitive mechanisms of Chinese is not only an urgent need to fill the screening gap for tone languages, but also an indispensable foundation for future robust cross-lingual domain adaptation.

**Figure 1:**
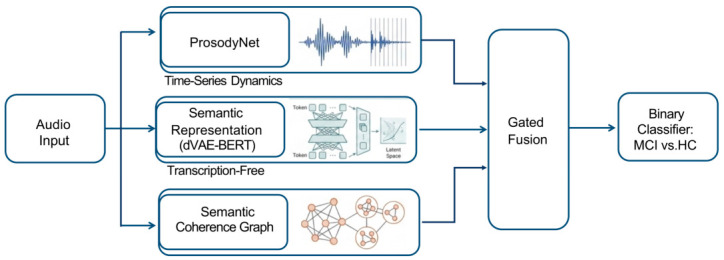
Overall architecture of the proposed Multi-Spec MCI-Net framework.

To address these issues and bridge the aforementioned gaps, this study proposes Multi-Spec MCI-Net, a multimodal framework that integrates prosodic features, discrete semantic representations, and token-level semantic coherence graphs through gated fusion (Figure 1). Experiments are conducted on the Chinese NCMMSC2021_AD dataset and the Mandarin/Chou subset of DementiaBank [30]. Ablation experiments and fusion weight analysis are further used to evaluate the contribution of each modality and improve interpretability.

The main contributions of this study are as follows: (1) a deep multimodal fusion framework for HC/MCI classification; (2) token-based semantic coherence graph modeling; (3) fine-grained prosodic temporal modeling; (4) staged optimization for small-sample and imbalanced data; and (5) systematic validation on Chinese speech datasets, establishing a robust benchmark for tone-language cognitive screening.

## 2. Materials and Methods

**Figure 2:**
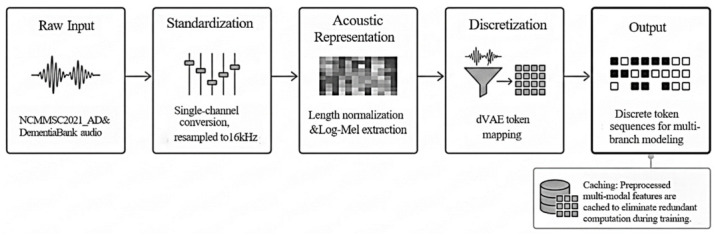
Acoustic Data Pipeline and Discretization.

This section delineates the technical implementation of the proposed framework. Figure 2 illustrates the acoustic data-preprocessing pipeline, encompassing raw waveform extraction, spectral feature transformation, and discretization strategies to generate structured multimodal representations for downstream fusion.

### 2.1. Data Source and Sample Characteristics

The experiments were conducted on the NCMMSC2021_AD Chinese speech challenge dataset and the Mandarin/Chaozhou subset of DementiaBank [30]. The task was formulated as binary classification of healthy control (HC) versus mild cognitive impairment (MCI). The NCMMSC2021_AD dataset comprises 320 samples in total, partitioned into 201 training and 119 test samples. To enhance data diversity, the Chaozhou subset was split 1:1 into training and test portions and merged with NCMMSC2021_AD, yielding a combined training set of 168 HC and 165 MCI samples, and a combined test set of 105 HC and 108 MCI samples.

All recordings were elicited via a picture-description task and stored as monophonic 16 kHz 16-bit WAV files.

### 2.2. Preprocessing and Feature Caching

All speech signals were converted to monophonic waveforms and resampled to 16 kHz. A WebRTC-based voice activity detection (VAD) algorithm was applied with a cognitively informed silence-filtering protocol—only non-speech segments exceeding 2 s (typically environmental noise) were discarded, whereas all unfilled pauses under 2 s were retained to preserve pathologically salient markers of cognitive load and lexical retrieval latency.

The resulting variable-length signals underwent short-time Fourier transform, followed by Mel-filterbank decomposition with 128 Mel bins and logarithmic compression to an approximately 80 dB dynamic range, yielding high-resolution time-frequency representations. The extracted Log-Mel spectrograms assumed the shape [128, time_steps], where *time_steps* constituted a variable, sample-dependent temporal dimension rather than a fixed constant. These non-uniform sequences were subsequently processed by a temporal attention pooling mechanism that performed adaptive aggregation, enabling seamless end-to-end handling of variable-length inputs while eliminating the clinical assessment bias inherent in fixed-length preprocessing paradigms.

To improve efficiency and reproducibility, prosodic features, dVAE-BERT inputs, and graph inputs were pre-extracted and cached offline.

### 2.3. Multimodal Feature Construction

The prosodic branch uses 1 s sliding windows with 0.5 s stride to generate 24-dimensional acoustic descriptors, preserving local temporal dynamics. The semantic branch encodes log-Mel spectrograms into discrete tokens with dVAE and then applies BERT to obtain higher-level representations. The graph branch constructs a token-level semantic coherence graph using temporal edges and cosine-similarity-based semantic edges, which is then processed by a GCN.

#### 2.3.1. Audio Preprocessing

Let the original audio signal be x(t), discretized as $\{x[n]{\}}_{n=0}^{N_{s}-1}$, where $N_{s}$ is the number of samples and $f_{s}$ is the sampling rate. Each waveform is first converted to mono and, if necessary, resampled to $f_{s}$ = 16 kHz. The signal is then normalized to a fixed length $T_{0}=60\;s$ by truncation or zero padding.

A short-time Fourier transform (STFT) is applied, followed by Mel-filterbank projection and log transformation, producing a 128 ×L log-Mel representation for dVAE input, where L denotes the number of Mel time frames. The configuration includes n_mels = 128, win_length = 400, and hop_length = 160, corresponding to a 25 ms frame length and 10 ms frame shift. The Mel-frame length is given by:

$$L\;=\left\lfloor \frac{N_{s}-\mathrm{win\_length}}{\mathrm{hop\_length}}\right\rfloor +1.$$

This preprocessing ensures that the input dimensions remain consistent across samples.

#### 2.3.2. Multimodal Feature Pre-Extraction and Caching

To mitigate redundant computation during training, an offline pre-extraction strategy with runtime loading is employed. For each sample, three feature modalities are pre-computed and cached: (1) prosodic temporal descriptors; (2) dVAE-BERT input sequences; and (3) semantic graph node features with corresponding adjacency matrices. This caching mechanism substantially reduces I/O overhead and enhances experimental reproducibility.

#### 2.3.3. Prosodic Temporal Features

Instead of using only global summary statistics, the prosodic branch performs sliding-window temporal modeling. Each waveform is segmented with a 1 s window and 0.5 s stride. For each window, 24-dimensional descriptors are computed, including RMS energy, zero-crossing rate, spectral centroid, spectral bandwidth, spectral contrast, and MFCC statistics. The resulting sequence is denoted as:

$$P\in R^{T\times 24}.$$

where T is the number of windows. In the original implementation, the sequence is truncated or padded to max_time_steps = 100. This design preserves local temporal patterns such as pauses, energy changes, and spectral variation.

#### 2.3.4. dVAE Discretization and Semantic Representation

Given the log-Mel spectrogram $M\in R^{128\times L}$, the dVAE encoder processes Mel frames sequentially and outputs a discrete index sequence through convolutional encoding and Gumbel-Softmax quantization. The codebook size is K = 256, and the vocabulary size becomes 260 after adding special symbols such as PAD, CLS, SEP, and MASK. The resulting sequence is:

$$z=[\mathrm{CLS},z_{1},\ldots ,z_{n},\mathrm{SEP}],$$

which is then fed to a BERT encoder with 6 Transformer layers, 12 attention heads, and hidden size 768. Unlike standard text BERT, the input here comes from discrete acoustic tokens rather than manual transcripts, allowing a unified semantic encoding path. This dVAE-BERT pipeline is based on the method proposed by Chen et al. [31], which converts continuous acoustic signals into text-like discrete sequences to leverage the contextual modeling capabilities of pre-trained language models.

#### 2.3.5. Token-Level Semantic Coherence Graph

For graph construction, N denotes the number of valid tokens retained after removing CLS/SEP. Let the token embeddings be $h_{i}\in R^{768}$, with d =768 as the BERT hidden size. Two types of edges are used: (1) temporal edges, where $\left|i-j\right|\leq d_{max}$ (default $d_{max}=10$), and (2) semantic edges, where cosine similarity $cos\left(h_{i},h_{j}\right)\geq \theta$ (default θ =0.5).

The resulting node feature matrix and adjacency matrix are:

$X\in R^{N\times 768}$,

$A\in \{0,1{\}}^{N\times N}$.

This token-level graph is processed by a graph convolutional network to capture semantic coherence and local discourse continuity.

### 2.4. Model Architecture

**Figure 3:**
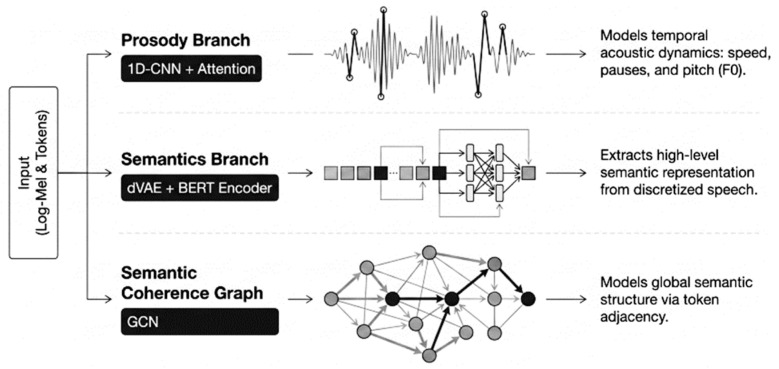
Schematic illustration of the Multi-Spec MCI-Net framework.

Multi-Spec MCI-Net comprises three specialized branches: ProsodyNet for temporal prosodic encoding, HierarchicalAttentionBERT for discrete semantic representation learning, and SemanticCoherenceGCN for graph-structured discourse modeling. Each branch projects its respective modality into a unified 256-dimensional latent space. These branch-specific embeddings are subsequently integrated via an adaptive gated fusion module, which learns sample-specific modality weights, and are passed to a two-layer multilayer perceptron for binary classification, as shown in Figure 3.

#### 2.4.1. Prosodic Branch

The input $P\in R^{B\times T\times 24}$ is reshaped to [B,24,T] for ‘Conv1d’ processing. Three multi-scale convolutions with kernel sizes 3, 5, and 7 are used to capture local temporal patterns, followed by dimensional reduction. Temporal attention is then applied, and the final output is projected to a 256-dimensional prosodic vector.

**Figure 4:**
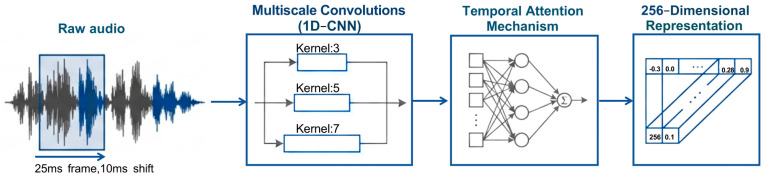
Network architecture of ProsodyNet for time-series prosodic modeling.

Figure 4 illustrates ProsodyNet, which extracts 24-dimensional prosodic features (pauses, F0, speech rate, and energy) and employs a 1D-CNN with Temporal Attention to capture multi-scale temporal dynamics of hesitation and pacing, circumventing the information loss associated with global averaging.

#### 2.4.2. Semantic Branch

**Figure 5:**
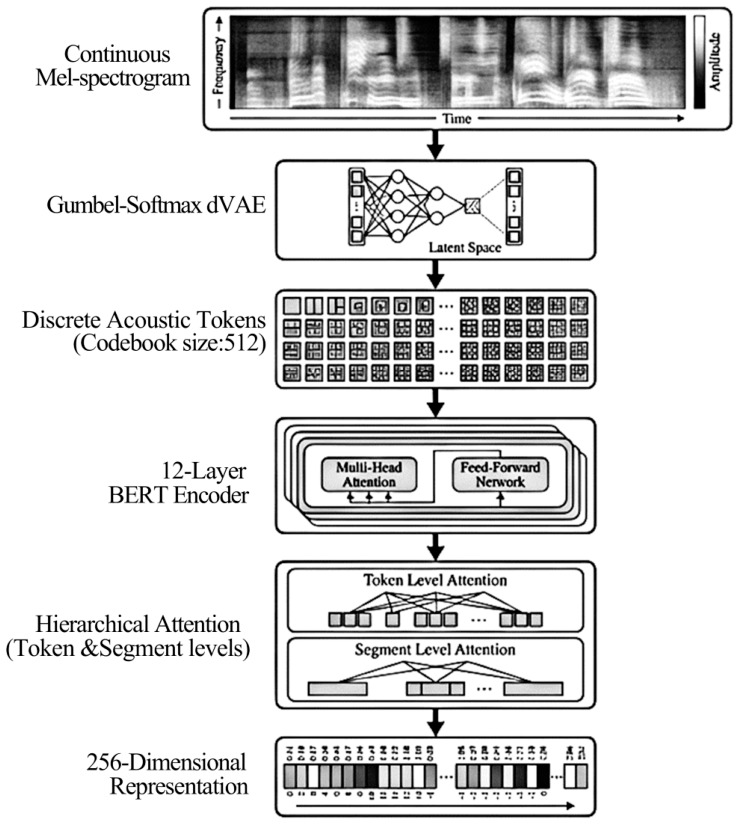
Discrete semantic representation module based on dVAE-BERT.

The semantic branch consists of a BERT encoder and hierarchical attention (Figure 5). It first produces a sequence representation:

$$H=\left[h_{1},\ldots ,h_{n}\right],h_{i}\in R^{768}.$$

Then a two-level aggregation is performed: token-level attention learns token importance, and segment-level attention divides the sequence into 8 segments and learns segment weights. The final output is a 256-dimensional semantic vector.

#### 2.4.3. Graph Branch

The graph branch is based on a three-layer graph convolutional network. After normalization of the adjacency matrix, the following update is applied:

$$H^{\left(l+1\right)}=\sigma \left(\hat{A}H^{\left(l\right)}W^{\left(l\right)}\right),$$

where A is the adjacency matrix, $H^{\left(l\right)}$ is the node feature matrix at layer l, $H^{\left(0\right)}=X$, $W^{\left(l\right)}$ is the weight matrix, and σ is a nonlinear activation. Node representations are pooled into a 256-dimensional graph vector, which captures structural coherence information.

#### 2.4.4. Gated Fusion and Classifier

The three branch vectors $p,s,g\in R^{256}$ are input to a gated fusion module to obtain weights:

$$\left[w_{p},w_{s},w_{g}\right]=\mathrm{softmax}\left(f([p;s;g])\right).$$

The fused representation is:

$$f=w_{p}p+w_{s}s+w_{g}g.$$

It is then passed through two fully connected layers (256 → 128 → 2) to produce logits.

#### 2.4.5. Adaptive Gated Fusion Mechanism

(1) Mathematical Formulation of Gated Fusion

The features extracted from the three modalities are projected onto a shared hidden dimension *d* (where *d* = 256 in this study), denoted as vectors $H_{p},{\;H}_{s},{\;H}_{g}\in R^{d}$.

First, a channel-wise concatenation is performed to construct a joint feature vector $H_{concat}$ that encapsulates the global multimodal context:

$$H_{concat}=\left[H_{p}⊕H_{s}⊕H_{g}\right]\in R^{3d}.$$

The concatenated features are then fed into a compact feedforward gating network with nonlinear activation to evaluate the relative importance of each modality for the current sample. An adaptive gating weight vector $g\;=[g_{p},{\;g}_{s},g_{g}]$ is generated via Softmax normalization:

$$g\;=\mathrm{Softmax}\left(W_{g}\cdot \mathrm{ReLU}(W_{h}H_{concat}+b_{h})+b_{g}\right),$$

where $W_{h}\in R^{d_{h}\times 3d}$ and $W_{g}\in R^{3\times d_{h}}$ are learnable weight matrices, $b_{h}$ and $b_{g}$ are bias terms, and ReLU introduces nonlinearity into the representation. The constraint $\sum_{i\in \{p,s,g\}}g_{i}=1$ ensures the convex combination property of the fusion.

**Figure 6:**
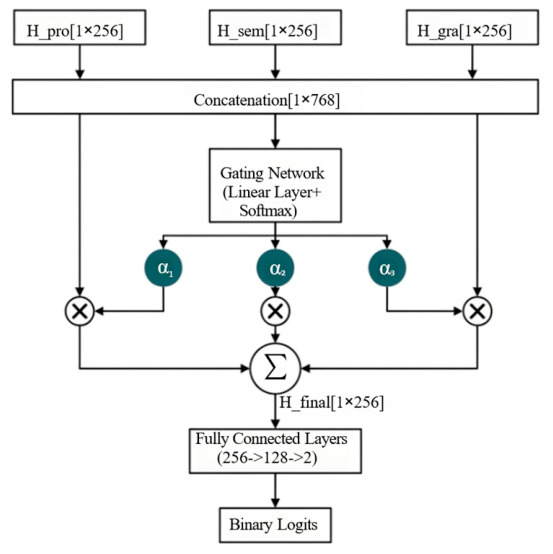
Micro-Topology: The Gated Fusion Mechanism.

Finally, the fused multimodal feature $H_{fused}\in R^{d}$ is obtained through element-wise scalar multiplication and vector addition:

$$H_{fused}={\;g}_{p}H_{p}+g_{s}H_{s}+g_{g}H_{g},$$

which is subsequently passed to the final classifier for MCI risk prediction, as shown in Figure 6.

(2) Gradient Regulation and Modality Dominance Prevention

The BERT-based semantic branch carries strong pre-trained priors. Under direct concatenation or simple averaging fusion, its high-dimensional representations produce disproportionately large gradients during backpropagation that readily overwhelm the substantially smaller gradients from the prosodic (1D-CNN) and graph (GCN) branches. This drives the model to collapse into a unimodal text classifier, thereby undermining the multimodal objective.

The adaptive gating mechanism addresses this through explicit gradient modulation via dynamic weights $g_{i}$. For any modality branch *i*∈{*p*, *s*, *g*}, the back propagated gradient is strictly regulated as:

$$\frac{\partial L}{\partial H_{i}}=g_{i}\cdot \frac{\partial L}{\partial H_{fused}}+\frac{\partial g_{i}}{\partial H_{i}}\cdot \frac{\partial L}{\partial g_{i}}.$$

During training, the gating network learns that semantic features possess relatively weaker discriminative power for early MCI detection, and actively suppresses the semantic weight $g_{s}$ while amplifying the prosodic weight $g_{p}$ and graph weight $g_{g}$. This effectively imposes damping on the frequently updated semantic features, thereby preserving independent optimization spaces for the prosodic and graph branches. Consequently, the complementary discriminative potentials—specifically the “discourse jumps” captured by the graph branch and the “temporal articulatory delays” captured by the prosodic branch—are fully exploited at the fusion stage, ultimately achieving a high screening efficacy.

### 2.5. Training Strategy and Evaluation

The main model is trained in three stages: freezing the semantic encoder, joint fine-tuning, and class-weighted optimization for MCI. Adam is used with branch-specific learning rates, gradient clipping, and learning rate scheduling. Ablation models use a simpler 20-epoch setting with Adam and a learning rate of $10^{-4}$.

Evaluation uses accuracy, recall, precision, F1–Score, and ROC AUC. Thresholds from 0.10 to 0.95 are scanned in steps of 0.05, and τ = 0.55 is selected as the operating threshold.

The primary evaluation encompasses threshold-sensitivity analysis, performance comparison between Multi-Spec MCI-Net and the dVAE-BERT baseline, confusion matrices, ROC curves, and prediction probability distributions. Ablation experiments systematically compare four model configurations—baseline (semantic branch only), semantic + prosody, semantic + graph, and the full multimodal architecture—under an identical threshold setting. Additionally, fusion weight analysis is employed to quantify the relative diagnostic contribution of each modality branch.

## 3. Results

### 3.1. Primary Evaluation of Multi-Spec MCI-Net

#### 3.1.1. Training Protocol and Checkpoint Selection

The main model was trained using the three-stage strategy detailed in Section 2: first, the semantic encoder was partially frozen to stabilize the non-semantic branches; subsequently, the full architecture was jointly fine-tuned; finally, the loss function was reweighted to prioritize the MCI class and reduce false-negative rates. Training proceeded for 50 epochs on the designated training set. Validation accuracy and F1–Score exhibited continuous improvement throughout optimization, with peak performance attained at approximately epoch 27. The training process monitored validation MCI F1 as the primary metric and retained a single best-performing checkpoint, thereby avoiding any test set contamination during model selection. Consequently, all reported test results were generated from a single forward pass using the fixed checkpoint under a predetermined threshold rule, ensuring an unbiased estimate of generalization performance.

#### 3.1.2. Classification Threshold Selection

**Figure 7:**
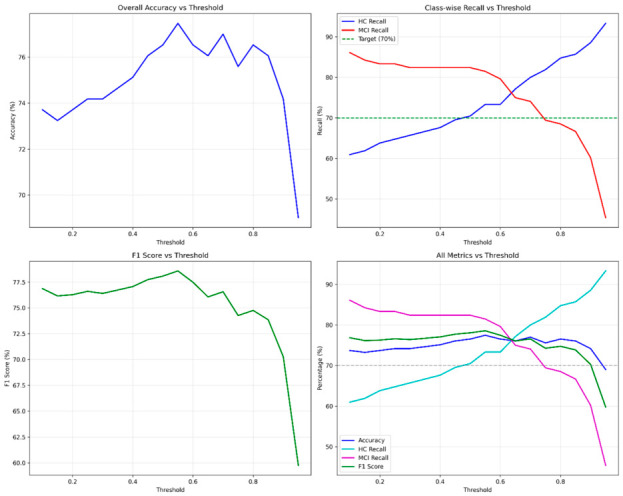
Threshold test results.

Binary neural networks output continuous probabilities, and the threshold τ divides the probability axis into two decision regions. Simply using the default threshold of 0.5 often fails to balance recall for HC and MCI, especially in imbalanced settings where the cost of missed diagnosis is relatively high. Therefore, this study scanned τ over {0.10, 0.15, …, 0.95} with a step size of 0.05 and obtained the optimal threshold of 0.55. This is shown in Figure 7 and Table 1. All main tables below are reported under τ = 0.55.

Under this threshold, Multi-Spec MCI-Net was evaluated on both the combined cross-dataset test set and the NCMMSC2021_AD competition hold-out set, with its performance benchmarked against the reproduced dVAE-BERT baseline. The comparative results are summarized in Table 2.

#### 3.1.3. Overall Performance on the Combined Competition + Clinical Test Set

**Figure 8:**
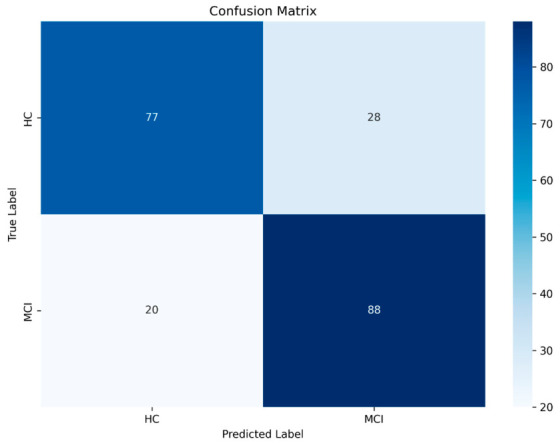
Confusion matrix of the multimodal model.

**Figure 9:**
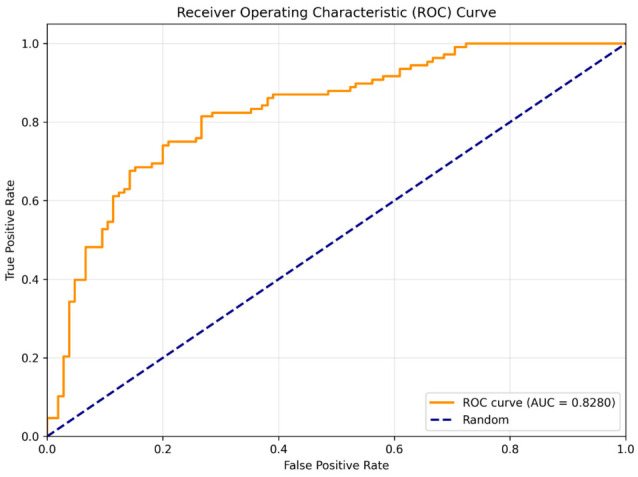
ROC curve.

**Figure 10:**
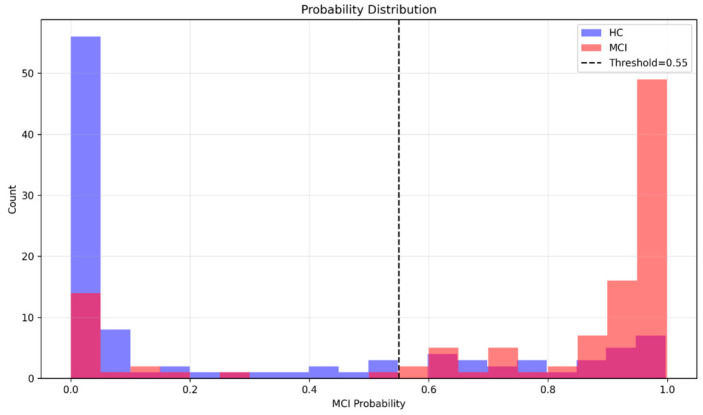
Prediction probability distribution.

Because the clinical data are more diverse and heterogeneous, evaluation on the combined dataset is more challenging. To better reflect the model’s robustness, the multimodal model was evaluated on the combined competition-plus-clinical test set, and the corresponding confusion matrix, ROC curve, and prediction probability distribution were analyzed, the results are shown in Figure 8, Figure 9 and Figure 10.

**Figure 11:**
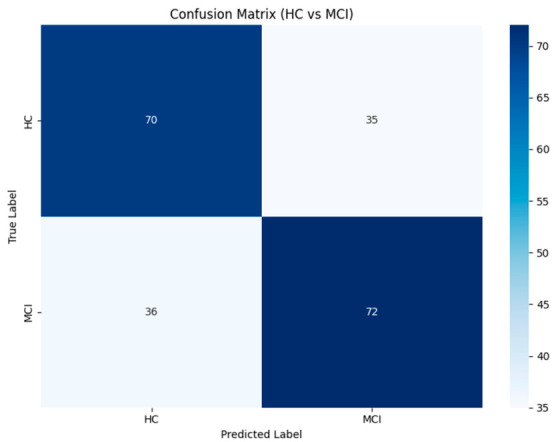
Confusion matrix of the ‘dVAE-BERT’ model.

**Figure 12:**
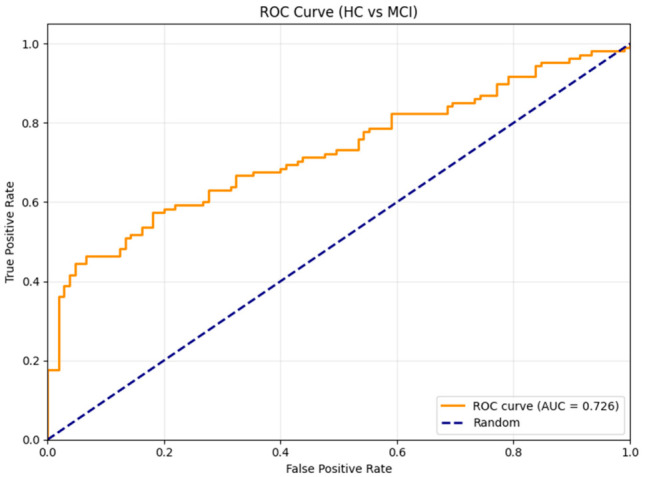
ROC curve of the ‘dVAE-BERT’ model.

To better demonstrate the improvement brought by the multimodal fusion model, the dVAE-BERT-based Alzheimer’s detection method was independently reproduced and adapted to the binary HC/MCI classification task. This reproduced baseline was evaluated on an identical held-out test set under the same experimental protocol, enabling direct, fair comparison. The corresponding confusion matrix and ROC curve are presented below in Figure 11 and Figure 12.

Overall, the 77.46% accuracy and 0.8280 ROC AUC of the proposed model represent a substantial improvement over the semantic-only ‘dVAE-BERT’ baseline, with an accuracy gain of about 10.79 percentage points. The model shows stronger discrimination ability for HC and MCI. In addition, the result reflects the effect of the MCI-oriented cost-sensitive training strategy: while maintaining relatively high accuracy, the model achieved an MCI recall of 81.48%, which greatly reduced the missed-diagnosis rate. This suggests that, under the current feature and model settings, the model has strong ranking ability for HC/MCI discrimination.

#### 3.1.4. Performance Analysis on the Pure Competition Dataset

**Figure 13:**
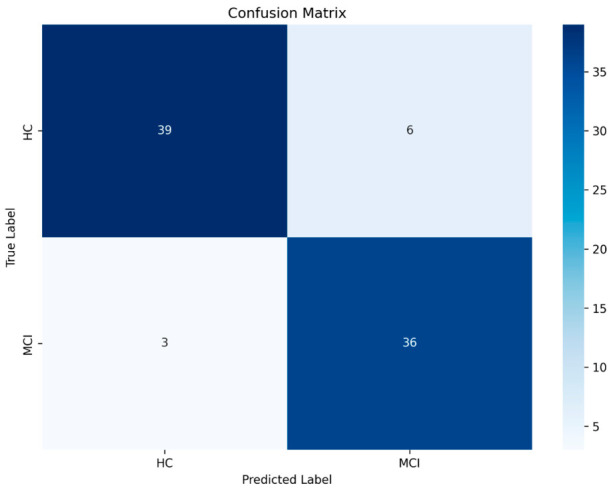
Confusion matrix of the model on the competition dataset.

**Figure 14:**
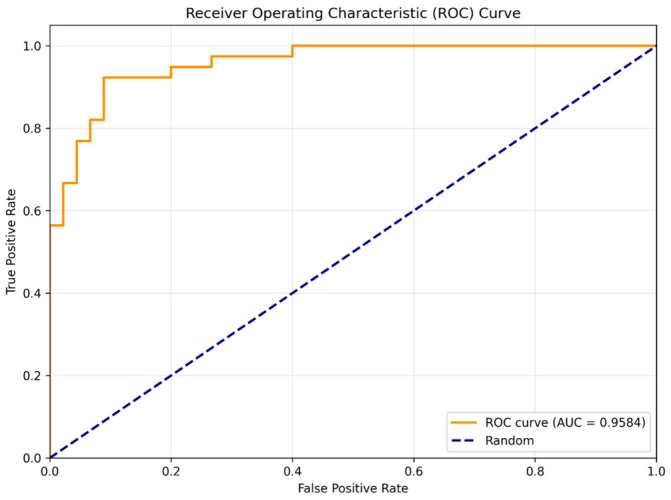
ROC curve on the competition dataset.

**Figure 15:**
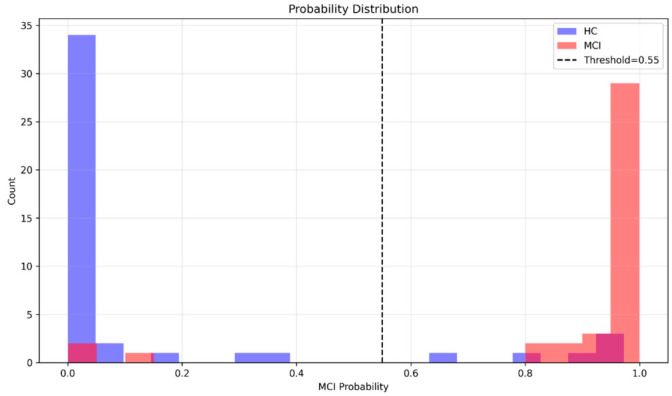
Prediction probability distribution on the competition dataset.

Owing to the relatively standardized acquisition protocol and stricter collection criteria governing the competition dataset, evaluation on this benchmark provides a controlled assessment of the model’s performance under more homogeneous conditions. The test results on the NCMMSC2021_AD dataset are presented below in Figure 13, Figure 14 and Figure 15.

As can be seen, the model achieves very high performance on the standardized competition dataset, with overall accuracy reaching 89.29%, MCI recall reaching 92.31%, and HC recall reaching 86.67%. This indicates that the model already has strong detection capability.

### 3.2. Ablation Experiments

#### 3.2.1. Experimental Setup

Four variants were evaluated: baseline (semantic branch only), ‘semantic_prosody’, ‘semantic_graph’, and ‘full’ (the same structure as the main model, i.e., three branches plus gated fusion). Ablation models were trained by ‘training/train_ablation.py’ for 20 epochs with Adam and a learning rate of 10^−4^. They were evaluated by ‘evaluation/evaluate_ablation.py’ on the same test set under τ = 0.55.

#### 3.2.2. Metric Comparison

The ablation results in Table 3 show that the semantic-only baseline achieves the highest HC recall at 80.95%, but its MCI recall is only 63.89%, which is far from the desired level. After adding the prosodic branch, MCI recall increases from 63.89% to 70.37%, an improvement of 6.48 percentage points. After adding the graph branch, the full model further improves MCI recall by 11.11 percentage points over ‘Semantic + Prosody’, and by 17.59 percentage points over the semantic-only baseline. Overall accuracy also increases by about 5.16%. These results indicate that multimodal fusion significantly reduces missed MCI cases and improves balanced classification performance.

Notably, the two-branch variants (Semantic + Prosody and Semantic + Graph) underperform the semantic-only baseline in accuracy (69.01% and 70.42% vs. 72.30%). This counterintuitive pattern arises from a gradient-scale mismatch inherent to naive feature concatenation. The semantic branch is built upon a pretrained BERT encoder with approximately 110 M parameters, generating substantially larger gradient magnitudes than the lightweight 1D-CNN prosodic branch (~50 K parameters) or the GCN branch. In the absence of the gated fusion mechanism (Section 2.4.5), simple concatenation of these heterogeneous representations causes the dominant BERT gradients to overwhelm the weaker signals from the auxiliary branches during backpropagation, effectively degrading the combined model into a noisier variant of the semantic-only baseline. The full three-branch model resolves this through the gating mechanism’s explicit per-modality gradient modulation, which regulates the contribution of each branch during training and prevents any single modality from dominating the optimization. This architectural insight—that adaptive gating is a prerequisite for effective multimodal fusion in small-sample regimes, rather than merely an optional refinement—is a key methodological contribution of this study.

### 3.3. Gated Fusion Weight Analysis

**Figure 16:**
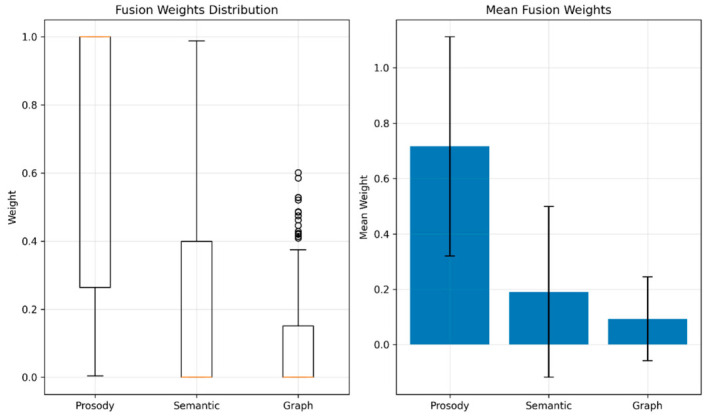
Fusion weights on the full test set.

**Figure 17:**
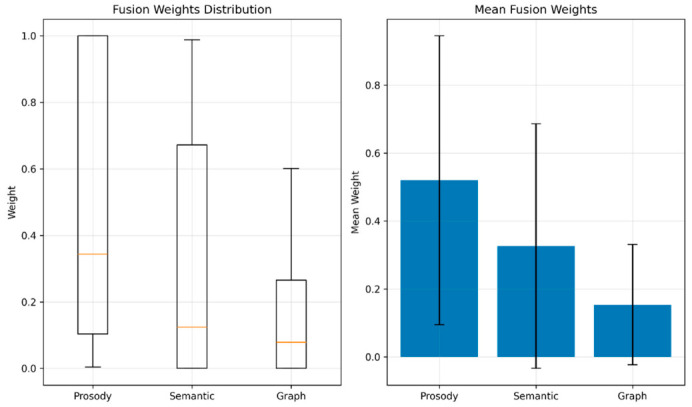
Fusion weights on the ‘NCMMSC2021_AD’ competition dataset.

During evaluation, fusion weights were returned so that three-dimensional weights for the prosody, semantic, and graph branches could be obtained for each test sample. The resulting ‘fusion_weights_analysis.txt’ file provides the fusion weights for the three branches. On the full test set, the average weights were 0.7160 ± 0.3959 for the prosodic branch, 0.1907 ± 0.3087 for the semantic branch, and 0.0933 ± 0.1514 for the graph branch. On the pure competition dataset, the corresponding values were 0.5197 ± 0.4248, 0.3264 ± 0.3598, and 0.1540 ± 0.1767, respectively. The according results are presented in Figure 16 and Figure 17 below.

Although the graph branch has the smallest average weight, the full model still outperforms the variants that remove individual branches, indicating that gated fusion dynamically assigns sample-level contributions rather than simply disabling one branch. This suggests that the graph branch still provides useful complementary information even when its average weight is relatively small.

## 4. Discussion

### 4.1. Benchmarking Against State-of-the-Art Methods

In the field of automatic cognitive-impairment detection, early studies predominantly relied on unimodal features. Acoustically, Xue et al. achieved 74.3% accuracy using CNNs on raw audio recordings [32], whereas Kumar et al. reached 87.6% with compact prosodic and voice-quality feature sets [33]. Textually, Karlekar et al. attained 83.84% on the DementiaBank corpus using CNN-LSTM architectures [34], later improved to 88.20% by Di Palo and Parde through the incorporation of psycholinguistic and affective features [35]. Despite continuous refinement, these unimodal approaches remain constrained by an information bottleneck that limits performance ceilings.

To overcome this limitation, multimodal fusion has emerged as the prevailing paradigm. Tóth et al. [36] and König et al. [37] established the feasibility of integrating automatic speech recognition (ASR) with semantic analysis; Gosztolya et al. combined acoustic and linguistic features to raise accuracy from the unimodal acoustic ceiling to 80–86% [38]; Fraser et al. further incorporated eye-tracking to reach 83% [39]. In recent deep-representation learning frameworks, Mahajan and Baths’s SpeechGRU [40] and Zhu et al.’s WavBERT [41]—which integrates Wav2Vec with BERT—both achieved approximately 83.1% accuracy. Nevertheless, most existing techniques still rely on simple feature concatenation or averaging, lacking adaptive decoupling of the complex cognitive compensatory mechanisms between macro-discourse topology and micro-temporal prosody.

By contrast, the proposed Multi-Spec MCI-Net breaks through these constraints. Relative to the aforementioned international baselines (which cluster in the 83–88% interval) and our own dVAE-BERT baseline, Multi-Spec MCI-Net achieves a significantly superior performance of 89.29% accuracy and an ROC AUC of 0.9584. Moreover, owing to its lightweight gated-fusion architecture and highly efficient 1D-CNN temporal attention network, the model attains high computational efficiency (~14 ms per sample) with a memory footprint strictly below 1 GB. Thus, without sacrificing inference speed, the framework delivers dual technical advantages in both screening accuracy and clinical deployability through the novel integration of semantic coherence graph topology and adaptive gating weights.

It is worth noting that recent studies have advanced AD/MCI research from complementary methodological perspectives that differ fundamentally from our speech-based multimodal fusion approach. Yu et al. [42] utilized multimodal DTI-ALPS and hippocampal microstructural signatures to reveal stage-specific pathways in AD progression—a neuroimaging paradigm that, while highly informative, remains dependent on specialized MRI scanners. Qin et al. [43] systematically evaluated the efficacy of virtual reality technologies on cognitive function in older adults with MCI, operating in the interventional rather than screening domain. Zhan et al. [44] focused on biomechanical analysis of traumatic brain injury, targeting acute structural injury rather than chronic neurodegeneration. Wang et al. [45] proposed Flat-Lattice-CNN for Chinese medical named entity recognition using written text rather than spoken audio. Hui et al. [46] investigated pharmacological mechanisms of chinonin in nervous system diseases at the molecular level. None of these works involve a multimodal cognitive impairment screening framework based on spontaneous Chinese speech, underscoring the unique contribution of the present study within the broader multi-level AD/MCI diagnostic ecosystem.

### 4.2. Cross-Modal Complementarity and Clinical Implications

Multi-Spec MCI-Net integrates prosodic, semantic, and semantic coherence graph features, demonstrating robust performance in mild cognitive impairment (MCI) detection. The results clearly illustrate complementarity among the three modalities. The semantic-only baseline exhibited a marked bias toward healthy controls (HC), yielding low MCI recall. Conversely, the incorporation of temporal prosody and graph-topological information substantially improved MCI sensitivity. This shift carries profound clinical significance: in early dementia screening, the clinical and temporal costs of false negatives (missed MCI cases) substantially outweigh those of false positives, as a missed diagnosis forecloses the narrow therapeutic window for early pharmacological and cognitive interventions.

Furthermore, the fusion weight analysis should not be interpreted as evidence of redundancy in the semantic or graph branches, despite the numerical dominance of the prosodic branch. Rather, it indicates that the gating module successfully learned sample-dependent adaptive contributions. The semantic coherence graph branch retains substantial value by providing word-level structural topology derived from the BERT pipeline, serving as a structural anchor when sequential semantic coherence begins to deteriorate.

### 4.3. Cognitive Mapping of Prosodic Features and Temporal Attention Attribution

To comprehensively capture subtle acoustic distortions in spontaneous speech among MCI patients, we constructed a comprehensive 24-dimensional prosodic feature space. This feature set was not assembled through empirical random stacking but was deeply grounded in established clinical speech-biomarker literature, encompassing three core cognitive-linguistic dimensions. First, temporal measures (e.g., pause rate, speech rate) directly reflect cognitive-motor planning delays and lexical access deficits arising from prefrontal executive dysfunction. Second, fundamental-frequency (F0) statistics (mean, standard deviation, range) map the early degradation of fine neural control over vocal-fold tension by the basal ganglia–cortical loop. Third, energy-contour features characterize underlying respiratory support and compensatory articulatory effort. These dimensions have been extensively validated as the most acoustically sensitive correlates of MCI.

In this study, we deliberately avoided aggressive single-feature elimination (e.g., recursive feature elimination) to quantify absolute individual contributions. This decision was motivated by two considerations. First, the primary objective was to validate the fusion efficacy of the multimodal architecture rather than to optimize unimodal feature engineering. Second, in small-sample medical scenarios, forced ablation of individual features disrupts the joint distribution of continuous acoustic parameters, readily compromising feature-space integrity and inducing model instability.

As a more globally informed alternative, the framework innovatively integrates a 1D-CNN with temporal attention in the prosodic branch. This architecture preserves the original joint distribution of acoustic features while adaptively assigning significantly higher attention weights to the most discriminative pathological signals—such as anomalous pause-rate fluctuations or F0 jitter—during training. Consequently, by extracting and analyzing the attention weight distribution matrix from the trained model, we can implicitly yet precisely infer the relative contributions of specific sub-features to the final classification. Future work, leveraging larger-scale multi-center longitudinal datasets, will incorporate SHAP (SHapley Additive exPlanations)-based fine-grained attribution analysis, treating feature-level selection as an independent extension to guide the development of more lightweight clinical tools.

### 4.4. Cognitive Mapping of Acoustic–Semantic Biomarkers

Previous studies often positioned deep learning frameworks as purely engineering-oriented classification solutions rather than as causal explanations of pathology. Our feature attribution analysis bridges this gap. By mapping the most discriminative computational features to established theories in cognitive neurolinguistics and speech motor control, we effectively transform the model from a “black box” into a clinically interpretable “grey box.”

(1) The model assigned exceptionally high weights to macro-prosodic features such as silent pause frequency and pause rate. From a cognitive perspective, speech production requires intensive prefrontal cortex (PFC) coordination of working memory alongside temporo-parietal involvement in semantic retrieval. In MCI, early executive dysfunction leads to lexical access deficits (“tip-of-the-tongue” phenomena). To maintain communication, the brain automatically adopts a “stalling strategy.” Multi-Spec MCI-Net precisely captures these non-syntactic silent pauses, quantifying them not merely as silence but as acoustic biomarkers of PFC cognitive overload.

(2) Beyond macro-pauses, the 1D-CNN temporal attention mechanism exhibits high sensitivity to micro-acoustic perturbations such as jitter (frequency instability) and shimmer (amplitude instability). Human phonation relies on sub-millisecond coordination of laryngeal muscles by the basal ganglia–cortical–cerebellar circuit. Neurodegenerative cascades (e.g., amyloid-β and tau accumulation) subtly disrupt these descending motor pathways even at the MCI stage. The model’s reliance on jitter and shimmer variance indicates that it detects subclinical vocal-fold vibration instability—essentially performing an “acoustic biopsy” of early neuromuscular decline.

(3) In specific MCI samples, counterintuitive surges in the energy coefficient of variation (Energy CV) and F0 range correlated strongly with topological sparsity detected by the semantic graph branch. This reflects a cognitive–articulatory compensatory mechanism: when patients struggle with semantic retrieval or topic maintenance, cortical arousal elevates. Once the target lexical item is retrieved, concomitant transient sympathetic excitation causes excessive subglottal pressure, manifesting as exaggerated articulatory effort or misplaced stress. The gating fusion mechanism seamlessly learned this cross-modal rule: low semantic coherence frequently triggers high-energy acoustic bursts.

### 4.5. Cross-Modal Cognitive Coupling Between Prosody and Semantic Coherence

From a cognitive-neuroscience perspective, speech production is a highly orchestrated neural process involving left frontotemporal networks for semantic retrieval and right-hemisphere–subcortical circuits for prosodic motor execution. MCI, as a heterogeneous syndrome, does not confine pathological damage to isolated neural circuits; rather, it affects semantic memory and executive-function systems to varying degrees across patients. This neuroanatomical foundation provides the biological premise for cross-modal coupling between prosodic features and semantic coherence.

Specifically, MCI patients, impaired in prefrontal executive function and temporal-lobe semantic memory, face lexical-access difficulties. When patients encounter “tip-of-the-tongue” states or semantic network retrieval failures during picture description or spontaneous conversation, their cognitive system initiates a compensatory monitoring mechanism: the PFC increases processing time to trade for retrieval success probability. This underlying process manifests at the acoustic surface as strategic pause prolongation, speech-rate reduction, and abnormal F0 contour regulation. In other words, semantic coherence breakdown (topological sparsity captured by the graph branch) and prosodic temporal distortion (pause/rate anomalies captured by the prosodic branch) are not independent parallel symptoms but rather mappings of the same neurocognitive insult at different representational levels. “Break points” in the semantic network trigger executive delays in the PFC–basal ganglia–cerebellar motor loop, while dynamic prosodic perturbations serve as the acoustic “signature” of semantic retrieval difficulty.

Our ablation data provide empirical support for this theoretical coupling. As shown in Table 3, with semantic and graph features alone (Semantic + Graph), the model achieved 70.42% accuracy and 75.00% MCI recall. Upon integrating temporal dynamic prosodic features via gated fusion (Full Model), accuracy improved to 77.46% and MCI recall increased to 81.48%. This performance improvement is not a mere feature superposition effect but reflects synergistic representations of complementary facets of cognitive impairment: the semantic graph branch captures lexical-conceptual network disintegration, while the prosodic branch captures executive-motor compensatory delay. Together, they constitute a complete cognitive acoustic portrait of MCI speech degradation.

The adaptive weight allocation of the gating mechanism further reveals the inter-individual variability of this coupling. In some samples, prosodic weights significantly exceed semantic weights, suggesting that these patients may predominantly suffer from executive-motor pathway involvement (e.g., subcortical small vessel disease MCI); in others, semantic graph weights rise relatively, implicating more prominent temporal-lobe semantic network degeneration (e.g., amnestic MCI). This sample-specific modality weight distribution essentially constitutes a data-driven subtyping of MCI heterogeneity, offering clinicians fine-grained cognitive phenotypic information beyond binary classification.

In summary, the prosodic and semantic coherence branches are not isolated technical modules but deeply coupled through the causal chain of neurocognitive impairment: semantic retrieval difficulty → executive monitoring compensation → prosodic motor delay. This cross-modal coupling mechanism not only explains the intrinsic logic of the performance leap observed in ablation experiments but also provides the theoretical foundation for transforming Multi-Spec MCI-Net from a “black-box” classifier into a “grey-box” clinical decision-support tool. Future research will incorporate longitudinal follow-up data to validate the association between prosody–semantic weight ratios and MCI subtype conversion trajectories, exploring the prognostic value of this coupling in disease-progression prediction.

### 4.6. Theoretical Trade-Offs of Fusion Mechanisms and Architectural Scalability

Although the gating fusion module adopted in this study is mathematically concise, this design reflects deliberate theoretical trade-offs for small-sample clinical scenarios. Across the spectrum of current multimodal-fusion paradigms, conventional feature concatenation is overly rigid, failing to capture dynamic nonlinear relationships among modalities. Conversely, cutting-edge Transformer-based cross-modal attention encoding can explicitly mine fine-grained temporal-semantic alignment through computationally intensive Query–Key interaction matrices; however, their vast parameter spaces on limited clinical datasets such as NCMMSC2021_AD (320 samples total) readily induce severe dimensional catastrophe and overfitting risks.

In light of this, we explicitly characterize our gating fusion mechanism as a “low-rank regularized attention approximation tailored for small-sample clinical scenarios.” It essentially strips away costly frame-level or word-level dot-product computations, instead mapping concatenated features to a compact low-dimensional probability space through an extremely lightweight single-layer feedforward network. This “restrained” fusion paradigm retains the core attention concept—sample-level dynamic weight allocation according to individual patient symptoms—while imposing strong low-rank parameter regularization constraints. Thereby, it ensures classification accuracy while effectively curbing deep-network memorization of small-sample clinical noise.

More importantly, Multi-Spec MCI-Net adopts a highly modular architectural design. The current gating module serves as an efficient “plug-and-play” component under low-resource conditions. We fully acknowledge the potential of deep cross-modal attention mechanisms to enhance interpretability. Because the framework’s feature extraction branches are fully decoupled, future acquisition of larger-scale multi-center clinical datasets will permit seamless upgrading—direct replacement of the current gating module with deep cross-modal attention modules (e.g., using temporal prosodic features as Query to guide weighted extraction of graph-node features). This forward-looking design confers strong technical scalability, enabling continuous evolution alongside the accumulation of real-world clinical data.

### 4.7. Clinical Translational Value and Digital Biomarker Validation

The ultimate medical objective of this study is not merely to pursue algorithmic accuracy extremes but to provide a low-cost, non-invasive, large-scale early-screening tool for community and home settings. Conventional AD/MCI diagnosis relies heavily on neuroimaging (MRI/PET) or invasive biomarker assays (e.g., lumbar puncture), rendering population-wide screening impractical in resource-limited community settings. By contrast, Multi-Spec MCI-Net requires only lightweight spontaneous-speech recording, offering instantaneous inference and low resource consumption. This aligns with the practical medical demands of population-level screening—high throughput, low barrier, and easy accessibility—providing technical feasibility for daily cognitive-health monitoring on home smart terminals.

To further establish a substantive medical link between the model’s algorithmic output and current clinical gold standards, future work will conduct in-depth clinical quantitative evaluation during validation. Leveraging clinical metadata available in the NCMMSC2021_AD dataset (HC mean MMSE = 28.5 ± 1.2; MCI mean MMSE = 25.3 ± 2.1), we will perform quantitative correlation analysis between the model’s final output—MCI risk probability—and subjects’ actual Mini-Mental State Examination (MMSE) scores. This analysis aims to reveal whether a significant negative correlation exists between predicted risk probability and MMSE scores reflecting overall cognitive level. Such correlation is crucial, as it demonstrates that the prosodic anomalies and discourse-jump features extracted by this multimodal framework are not abstract mathematical vectors detached from clinical reality but are digital biomarkers that genuinely map to and characterize the degree of patient cognitive decline.

Furthermore, future work must incorporate prospective clinical trials or clinician-in-the-loop validation. While Decision Curve Analysis (DCA) provides a rigorous methodology for quantifying the net clinical benefit of diagnostic strategies across varying threshold probabilities, its implementation is contingent upon prospective clinical intervention parameters that are not yet available in this algorithmic baseline study. Specifically, DCA requires: (a) disease prevalence in the target screening population; (b) relative cost-effectiveness ratios of false positives versus false negatives, reflecting the differential clinical consequences of missed diagnoses versus unnecessary referrals; and (c) patient-specific or policy-level threshold preferences that balance sensitivity and specificity according to healthcare resource constraints. These parameters necessitate real-world data from prospective community screening cohorts or memory clinic referral pathways, which lie beyond the scope of the present retrospective validation.

Once prospective clinical data are acquired through ongoing multi-center collaborations, we will implement DCA in three sequential stages: (1) parameter estimation, deriving population-specific disease prevalence and cost ratios from screening cohorts and clinician surveys; (2) curve generation, computing net benefit curves across the full threshold probability spectrum using the model’s predicted risk probabilities; and (3) clinical decision optimization, identifying threshold ranges that maximize net benefit for distinct clinical contexts (e.g., community-based mass screening versus specialist referral confirmation). This staged approach will enable patient-centered threshold selection that explicitly incorporates the higher clinical cost of missed MCI diagnoses—a priority underscored by our current threshold-sensitivity analysis.

In the present study, we have completed cost-sensitive validation via threshold-sensitivity analysis, demonstrating the model’s performance trade-offs across clinically relevant operating points. Formal DCA will be systematically conducted as an integral component of subsequent Stage-II prospective clinical validation studies, following the SPIRIT-AI/CONSORT-AI reporting framework for AI-interventional trials.

### 4.8. Ethics and Privacy Considerations

As artificial intelligence penetrates deeply into medical diagnostics, privacy protection and ethical governance for speech and cognitive health data have become non-negotiable prerequisites for clinical deployment. This study establishes rigorous ethical and privacy safeguards in both data usage and future deployment planning.

(1) The high-quality Chinese speech corpus (NCMMSC2021_AD) used in this study strictly adhered to international ethical guidelines such as the Declaration of Helsinki during original data collection and corpus construction. All recordings were conducted under the supervision and approval of Institutional Review Boards (IRBs) at collaborating medical institutions, and all subjects or their legally authorized representatives provided written informed consent. Critically, prior to release to algorithm researchers, the data underwent thorough de-identification and desensitization; all direct identifiers and sensitive metadata potentially revealing subject identity were rigorously removed, ensuring data compliance at the source for retrospective research.

(2) Spontaneous speech signals carry extremely high privacy sensitivity, bearing not only pathological features but also biometric identity, emotional states, and domestic acoustic environments. Regarding the prospect of deploying digital screening tools at scale in community and home settings, we considered edge-deployment feasibility and security from the outset of model design. Multi-Spec MCI-Net demonstrates excellent lightweight architectural advantages, with GPU memory consumption during full-modality inference strictly controlled below 1 GB. This renders the model fully capable of operating on mainstream consumer mobile devices such as smartphones and tablets.

Accordingly, future deployment will adopt a forward-looking privacy-preserving paradigm based on edge computing. Under this paradigm, audio recording, multi-dimensional acoustic feature extraction (e.g., prosodic branch features), and local semantic coherence graph construction will be performed entirely offline on the terminal device. The system adheres to a strict “audio-never-leaves-device” physical isolation principle. Subsequently, only feature vectors—high-dimensional feature vectors or final MCI risk probabilities processed through irreversible hashing and dimensionality reduction—are transmitted via encrypted channels to cloud medical-management back ends or physicians’ terminals.

This “edge computing, cloud collaboration” architectural design physically eliminates the risk of raw speech data interception, eavesdropping, or misuse during network transmission. While ensuring physicians receive precise cognitive screening metrics, it maximally safeguards elderly patients’ data privacy rights, laying the essential ethical foundation and clinical acceptability for large-scale real-world deployment of speech-based AI diagnostic tools.

## 5. Limitations and Future Directions

### 5.1. Dataset Limitations, Ideal Data Profile, and Cross-Domain Adaptation

The NCMMSC2021_AD and DementiaBank Mandarin/Chaozhou subsets relied upon in this study represent the largest publicly available Chinese speech corpora with rigorous diagnostic labels in the cognitive-impairment research field. Category labels (HC, MCI, AD) in both datasets were diagnosed by professional clinicians according to international standardized criteria. Furthermore, these datasets cover two complementary cognitive load elicitation tasks—spontaneous conversation and picture description—largely mitigating assessment bias from single-task designs.

This study has completed cross-testing on these two independent, publicly available Chinese speech datasets, which partially mitigates the limitations of single-dataset evaluation and enhances the reliability of our findings through heterogeneous task protocols (picture description versus spontaneous dialog). However, we must acknowledge that these datasets remain cross-sectional data collected in relatively controlled acoustic environments, limiting to some extent the model’s direct generalization to real-world, multi-center, and longitudinal clinical cohorts.

From algorithm engineering to real-world clinical decision support systems, validation framework applicability requires higher-dimensional data support. For the multimodal framework proposed herein, future “ideal datasets” should possess four key characteristics: (a) Longitudinal tracking capability—longitudinal audio recordings spanning subject evolution from HC to MCI to AD, to rigorously validate early-screening sensitivity decay and predictive validity; (b) Multi-center and acoustic heterogeneity—encompassing diverse mobile recording devices and complex everyday background noise, to test feature-extraction robustness; (c) Multidimensional biomarker alignment—rich annotations of neuropsychological scales (e.g., MoCA sub-scores) and physiological biomarkers (CSF, MRI), to support deeper pathological-mechanism correlation analysis; and (d) Demographic representativeness—representative across dialects, education levels, and socioeconomic status. Although such multi-center cohort datasets are currently under joint construction and not yet publicly available, they constitute the inevitable direction for future field development.

Despite current data constraints, it is important to emphasize that Multi-Spec MCI-Net’s architectural design is inherently dataset-agnostic. Its highly modular three-branch design enables seamless retraining on any well-annotated speech corpus without modifying underlying network topology. The framework’s current medical applicability should be rigorously understood as conditional on the distribution of existing Mandarin corpora.

When generalizing to other languages (e.g., the English ADReSS dataset), different dialect systems, or novel speech elicitation tasks, direct transfer often induces domain shift due to phonetic typological differences or acoustic environment biases. To address this, future work will employ unsupervised domain adaptation strategies for cross-linguistic expansion. Specifically, we will utilize maximum mean discrepancy or domain-adversarial neural networks to remove forcibly non-pathological feature shifts arising from language-specific articulatory habits or recording device characteristics in a shared high-dimensional latent space, thereby preserving pure acoustic representations of cognitive decline.

### 5.2. Overfitting Mitigation and Real-World Heterogeneity

Although the NCMMSC2021_AD dataset is highly representative in the clinical cognitive screening domain—comprising 320 samples (201 training, 119 test)—its relatively limited size poses potential overfitting risks for multimodal deep networks containing tens of millions of parameters. To mitigate this, we designed multiple stringent defense mechanisms in both algorithmic architecture and training paradigm.

First, a progressive three-stage training strategy was adopted. In Stage 1, we completely froze the large pretrained BERT encoder (98.2% of total parameters), optimizing only the lightweight prosodic branch and graph network. In Stage 2, joint fine-tuning was performed with an extremely small learning rate (1 × 10^−4^). This design effectively prevents deep network overfitting on small samples and catastrophic forgetting of pretrained knowledge. Second, high dropout rates (0.3) and weight decay (L2 regularization, 1 × 10^−5^) were applied across all feature extraction branches and the gating fusion layer, together with early stopping based on validation metrics (patience = 10), ensuring training termination at the point of optimal generalization.

Regarding real-world data heterogeneity, the picture description task underlying this dataset naturally requires subjects to perform autonomous language planning and information organization in complex visual scenes, providing rich cognitive load and spontaneous speech pattern diversity. However, when facing broader real-world dialect backgrounds, generalization performance warrants in-depth clinical investigation. On one hand, Multi-Spec MCI-Net demonstrates a foundation of cross-dialect robustness: the macro-features it relies upon (e.g., meaningless pause rate, speech rate) and the semantic coherence graph topology constructed from sentence-level embeddings reflect deep executive function and logical flow coherence. These high-level cognitive biomarkers are relatively robust to underlying dialectal accents or vowel shifts and are less susceptible to dramatic regional pronunciation habits. On the other hand, extreme dialectal differences may still introduce noise into micro-acoustic features (e.g., F0 perturbation) or ASR transcription accuracy. Thus, although the multimodal gating fusion mechanism buffers to some extent the vulnerability of single modalities (e.g., text semantics) to dialectal influence, future multi-center clinical trials remain urgently needed to construct heterogeneous cohorts encompassing multiple regions and dialectal accents, for comprehensive validation and fine-tuning of this screening framework’s real-world generalizability in community healthcare settings.

### 5.3. Cross-Lingual Generalization and Transfer Learning: A Prospective Outlook

To address the generalization constraints inherent to single-context training and validation on Mandarin Chinese data, future work will prioritize cross-linguistic applicability through systematic transfer learning. The cross-linguistic generalization experiments outlined below have not been conducted in the current study and are presented solely as a methodologically specified roadmap for future investigation. All experimental results reported in this paper are based on Mandarin Chinese speech data. Given fundamental typological differences in acoustic compensatory mechanisms between tonal languages (e.g., Mandarin) and stress-timed languages (e.g., English), we will validate Multi-Spec MCI-Net’s cross-linguistic robustness using internationally recognized AD speech corpora, specifically the ADReSS challenge benchmark and the English subset of DementiaBank.

Our technical approach centers on unsupervised domain adaptation with dual-objective feature alignment. By jointly optimizing multi-kernel maximum mean discrepancy (MK-MMD) and domain-adversarial training losses in a high-dimensional latent space, we aim to explicitly disentangle pathological acoustic biomarkers from non-pathological confounders—namely, language-specific articulatory habits, inter-ethnic vocal-tract physiological variation, and hardware-dependent acquisition artifacts. This decoupling strategy will enable precise quantification of the framework’s screening robustness across diverse linguistic and cultural contexts, thereby establishing clear algorithmic evidence and clinically actionable applicability boundaries for future medical deployment.

We emphasize that the present study prioritizes rigorous baseline validation within the Chinese tonal language context. Cross-lingual transfer learning and out-of-domain evaluation are deliberately reserved for independent, systematically designed follow-up studies to ensure methodological rigor and reproducibility.

### 5.4. Additional Limitations and Future Work

First, the current model exhibits limited cross-task and cross-linguistic transferability. Because Mandarin is a tonal language—its F0 trajectory is tightly bound to lexical semantics—the observed compensatory mechanisms may differ fundamentally from those in stress-timed languages such as English. Future work will introduce unsupervised domain adaptation and maximum mean discrepancy alignment to evaluate cross-linguistic robustness on datasets such as ADReSS. Second, potential modality imbalance exists in the gating fusion. To fully exploit the potential of the graph and semantic branches, future iterations will explore orthogonality constraints in the loss function to enforce modality decoupling, while introducing cross-modal attention mechanisms (e.g., using temporal prosody to guide semantic weighting). Finally, real-world clinical deployment requires dataset scale expansion and prospective testing on independent external clinical cohorts to rigorously validate generalizability and mitigate potential preprocessing biases.

## 6. Conclusions

This study presents Multi-Spec MCI-Net, a multimodal deep learning framework for automated HC/MCI classification from spontaneous Chinese speech. By jointly modeling temporal prosodic dynamics, discrete semantic representations, and token-level semantic coherence graphs within an adaptive gated fusion architecture, the proposed method captures complementary cognitive-linguistic biomarkers of early neurodegeneration. Extensive experiments on the NCMMSC2021_AD and DementiaBank Mandarin datasets demonstrate that the full multimodal configuration substantially outperforms unimodal baselines, achieving high sensitivity for MCI detection while maintaining strong specificity. Ablation studies and fusion weight analyses further corroborate that adaptive integration of heterogeneous speech modalities yields synergistic diagnostic gains beyond simple feature concatenation.

These findings establish that natural speech encodes robust, non-invasive digital biomarkers for early cognitive impairment screening. The framework offers a scalable, cost-effective alternative to conventional neuroimaging and biomarker assays, with immediate potential for deployment in community-based or primary-care settings where resource constraints preclude invasive diagnostics. Future research will prioritize cross-lingual validation on independent cohorts, expansion to diverse speech elicitation paradigms, and the incorporation of longitudinal tracking to ascertain the framework’s utility in monitoring disease trajectory and therapeutic response.

## Figures and Tables

**Table 1:** Threshold-Sensitivity Analysis.

τ	Accuracy	HC Recall (Specificity)	MCI Recall (Sensitivity)	F1–Score
0.10	73.71%	60.95%	86.11%	76.86%
0.15	73.24%	61.90%	84.26%	76.15%
0.20	73.71%	63.81%	83.33%	76.27%
0.25	74.18%	64.76%	83.33%	76.60%
0.30	74.18%	65.71%	82.41%	76.39%
0.35	74.65%	66.67%	82.41%	76.72%
0.40	75.12%	67.62%	82.41%	77.06%
0.45	76.06%	69.52%	82.41%	77.73%
0.50	76.53%	70.48%	82.41%	78.07%
0.55	77.46%	73.33%	81.48%	78.57%
0.60	76.53%	73.33%	79.63%	77.48%
0.65	76.06%	77.14%	75.00%	76.06%
0.70	77.00%	80.00%	74.07%	76.56%
0.75	75.59%	81.90%	69.44%	74.26%
0.80	76.53%	84.76%	68.52%	74.75%
0.85	76.06%	85.71%	66.67%	73.85%
0.90	74.18%	88.57%	60.19%	70.27%
0.95	69.01%	93.33%	45.37%	59.76%

**Table 2:** Test Set performance comparison of ‘Multi-Spec MCI-Net’.

Metric	Fusion Model on the Full Test Set	Fusion Model on the Competition Test Set	‘dVAE-BERT’ Model
Accuracy	77.46%	89.29%	66.67%
ROC AUC	0.8280	0.9584	0.7257
HC Recall	73.33%	86.67%	66.67%
MCI Recall	81.48%	92.31%	66.67%
F1–Score	78.57%	88.89%	66.98%

**Table 3:** Ablation results (τ = 0.55).

Variant	Accuracy	HC Recall	MCI Recall	F1–Score
Baseline	72.30%	80.95%	63.89%	70.05%
Semantic + Prosody	69.01%	67.62%	70.37%	69.72%
Semantic + Graph	70.42%	65.71%	75.00%	72.00%
Full Model	77.46%	73.33%	81.48%	78.57%

## Data Availability

The data used in this study is derived from public domain resources. The dataset is available at https://talkbank.org/dementia/ (accessed on 9 March 2026), https://media.talkbank.org/dementia/Mandarin/Chou (accessed on 9 March 2026).
